# The Association of Radial Artery Pulse Wave Variables with the Pulse Wave Velocity and Echocardiographic Parameters in Hypertension

**DOI:** 10.1155/2018/5291759

**Published:** 2018-12-02

**Authors:** Li-jie Qiao, Zhen Qi, Li-ping Tu, Yu-hang Zhang, Li-ping Zhu, Jia-tuo Xu, Zhi-feng Zhang

**Affiliations:** ^1^Basic Medical College, Shanghai University of Traditional Chinese Medicine, 1200 Cailun Road, Shanghai 201203, China; ^2^Ultrasonic Diagnosis Department, The First People's Hospital of Taicang Affiliated to Suzhou University, 58 South Changsheng Road, Taicang 215400, China; ^3^Physical Examination Center, The First People's Hospital of Taicang Affiliated to Suzhou University, 58 South Changsheng Road, Taicang 215400, China

## Abstract

This study aims at exploring the cardiovascular pathophysiological mechanism of TCM (traditional Chinese medicine) pulse by detecting the correlation between radial artery pulse wave variables and pulse wave velocity/echocardiographic parameters. Two hundred Chinese subjects were enrolled in this study, which were grouped into health control group, hypertension group, and hypertensive heart disease group. Physical data obtained in this study contained TCM pulse images at “Guan” position of the left hand, pulse wave velocity, and echocardiographic parameters. Linear and stepwise regression analysis was performed to assess the association of radial artery pulse wave variables with pulse wave velocity and echocardiographic parameters in the total population and in each different group. After adjusting for related confounding factors, decrease of t_1_, t_5_ and increase of h_1_, h_3_/h_1_ were statistically associated with arterial stiffness in the total population (P<0.05). Moreover, the correlation study in each group showed that the decrease of both t_3_ and h_5_ was also related to arterial stiffness (P<0.05). In terms of echocardiographic parameters, the height of dicrotic wave indicated by h_5_ was the most relevant pulse wave variable. For the health control, h_5_ was negatively associated with interventricular septal thickness (VST) and left ventricular posterior wall thickness (PWT) (P<0.05), while for the hypertension population and those with target-organ damage to heart, increase of h_5_ might be associated with decrease of ejection fraction (EF) and increase of all the remaining echocardiographic parameters especially for left ventricular end-systolic diameter (LVDs) and Left ventricular end-diastolic diameter (LVDd) (P<0.05). In conclusion, we found radial artery pulse wave variables were in association with the arterial stiffness and echocardiographic changes in hypertension, which would provide an experimental basis for cardiovascular pathophysiological mechanism of radial artery pulse wave variables.

## 1. Introduction

Hypertension, as a primary global metabolic risk factor, accounts for 19% of global deaths [1]. Moreover, its varied complications are responsible for more than half of the cardiovascular diseases. Therefore, WHO has stated that the prevention and control of hypertension is one of the most important ways to reduce deaths and disabilities from noncommunicable diseases [2]. The prevalence of hypertension continues to increase in developing countries, especially in China—the populous country. The latest nationwide survey in China conducted from 2012 to 2015 demonstrates that 23.2% (approximate to 244.5 million) of the Chinese adult population had hypertension according to the Chinese guideline. Moreover, 46.9% of them were aware of their condition, 40.7% were taking prescribed antihypertensive medications, and only 15.3% had controlled hypertension [3].

Pulse palpation or pulse diagnosis is one of the most important diagnostic tools in traditional Chinese medicine (TCM) and other related oriental medicine systems. The traditional pulse diagnosis depends mainly on the subjective judgement of the TCM practitioners, which largely hinders the development of TCM pulse diagnosis by its subjectivity and fuzziness. Thereby, considerable researches have been carried out recently to measure radial pulse objectively and automatically by means of modern techniques, such as varied pulse waveform acquisition platforms [4–8], pulse waveform preprocessing and feature extraction [9–13], and pulse wave classification [14, 15].

By describing the pulse state of radial artery, TCM pulse diagnosis is actually a direct reflection of the cardiovascular condition. Because hypertension is a major risk factor in the cardiovascular diseases, therefore a number of studies have explored the correlation between hypertension and TCM pulse diagnosis. It was reported that the mean height of main peak (Mm) and the height of main peak (h_1_) of hypertension group were both higher than those of the healthy group when calibrated in Cheok by automatic pulse analyzer with array piezoresistive sensor [16]. Similar study indicated that the maximum pulse amplitudes in the left Guan and right Guan were the factors most strongly associated with hypertension after adjusting for age and body mass index (OR= 2.006 on the left and OR = 2.504 on the right) [17], while in another research, the pulse wave variables and pulse types measured in Guan by DMP 1000 were compared between hypertension group and normal blood pressure group. Significant differences between these two groups were found in the main and secondary pulse type; however no specific differences were found between them in the field of pulse wave variables [18]. In addition, the pathophysiological mechanisms based on pulse wave velocity and hemodynamics in patients' pulse images with hypertension were studied, and it revealed that the superficial, strong, and fast pulse images were related to higher systolic and pulse pressures, PWV, and heart rate [19, 20]. Besides, pulse wave variables were associated with different Sasang constitution [21] and quality of life [22] in hypertension patients. The prediction models of hypertension based on the radial artery pulse images have been studied and they achieved an accuracy of about 80% by related machine learning methods [4, 23].

However, the exact associations between radial artery pulse wave variables and cardiovascular conditions in hypertension are still not fully defined. Therefore, in this study, pulse wave velocity (PWV) and echocardiography were used, respectively, as a marker of arterial stiffness and cardiac function. The purpose of this study was to investigate whether radial artery pulse wave variables are present in association with the arterial stiffness and cardiac function in hypertension, so as to provide an experimental basis for cardiovascular pathophysiological mechanism of radial artery pulse wave variables in TCM.

## 2. Methods

### 2.1. Study Population

200 Chinese subjects were enrolled in the study from April 2016 to March 2018, and they were all from the First People's Hospital of Taicang affiliated to Suzhou University.

For current study, in order to diminish the impact of age on the cardiovascular condition, 159 subjects aged between 45 and 75 years were recruited from the overall participants [24]. Inclusion criteria were complete data on pulse wave velocity (PWV), echocardiography, and radial artery pulse wave variable data. The recruited participants were divided into three groups, which included the health control group, the hypertensive group (with systemic hypertension as their unique risk factor), and the hypertensive heart disease group (with systemic hypertension and simultaneously with target-organ damage to the heart). For the health control group, we included asymptomatic nonsmoking patients, with normal BP values (systolic blood pressure was between 90 and 139mmHg and diastolic blood pressure was between 60 and 89mmHg), without any diagnosis history of various acute and chronic diseases, and without taking any medical treatments. Hypertension group was enrolled according to the Chinese guideline for hypertension (revised edition in 2014) [25]; the inclusion criteria were as follows: patients with confirmed diagnosis of hypertension (detected under the static state and during separate visits without any inducement); normotensive patients but with administration of antihypertensive medication. Hypertension was defined as systolic blood pressure*⩾*140mmHg or diastolic blood pressure *⩾*90mmHg, while hypertensive heart disease group included those hypertensive patients with confirmed diagnosis of cardiac remodeling or dysfunction [26], such as left ventricle hypertrophy, left atrial enlargement, aortectasis, systolic dysfunction, and diastolic dysfunction.

Exclusion criteria contained ① patients with hypertrophic cardiomyopathy, cardiac amyloidosis, and other causes of left ventricular hypertrophy; ② patients with congenital heart disease, valvular disease, pulmonary heart disease, pericardial effusion, and pleural effusion; ③ history or symptoms of chronic metabolic diseases such as diabetes mellitus, dyslipidemia; ④ history of pulmonary, hepatic, or renal disease; ⑤ history of varied malignancy disease, endocrine system disease, infectious diseases, and mental disorders; ⑥ obesity patients whose BMI *⩾*30 Kg/m^2^; ⑦ pregnant and lactating women; ⑧ history of smoking.

The Medical Ethics Committee of the First People's Hospital of Taicang affiliated to Suzhou University approved the study, and written informed consent was obtained from all included subjects according to the Declaration of Helsinki.

### 2.2. Data Collection

Patient metadata were collected (i.e., sex, age, weight, height, duration time of hypertension, and history of taking antihypertensive drugs). Physical examinations included blood pressure, pulse pressure, body mass index (BMI), brachial-ankle pulse wave velocity (ba-PWV), ankle-brachial index (ABI), and echocardiographic parameters. Ba-PWV is widely acknowledged as the gold standard of arterial stiffness measurements [27], while ABI is a noninvasive method to assess the patency of peripheral occlusive arterial disease [28]. Echocardiography is a commonly used technique which can detect the anatomical structures and functional states of the heart and large vessels, and it has a relatively reasonable interobserver and intraobserver reliability for visual assessment of most echocardiographic parameters such as global left ventricular function, normal, and akinetic segments [29]. The measured parameters in echocardiography included diameter of ascending aorta (AAO), aorta sinus department diameter (AODd), left atrial diameter (LAD), interventricular septal thickness (VST), left ventricular end-systolic diameter (LVDs), left ventricular end-diastolic diameter (LVDd), left ventricular posterior wall thickness (PWT), ejection fraction (EF), and E/A ratio. In this study, blood pressure, Ba-PWV, and ABI were measured via the commercially available digital automatic BP monitor and arteriosclerosis detector (Omron model, BP-203RPEIII, Japan). The echocardiographic parameters were acquired through the ultrasonic diagnostic equipment (iE Elite, American).

### 2.3. Radial Artery Pulse Recordings and Analysis

**Figure 1:**
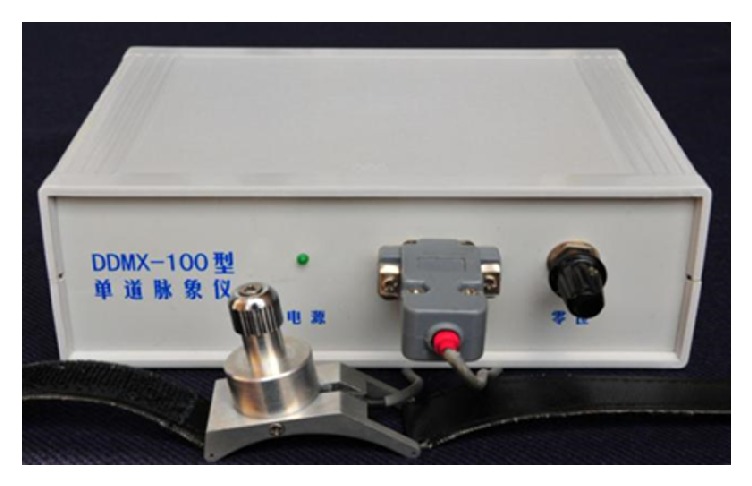
DDMX-100 type pulse measurement device.

Radial artery pulse images were recorded using a DDMX-100 type pulse measurement device which is developed by Shanghai University of Traditional Chinese Medicine (see Figure 1). The whole manipulation was conducted by the same trained practitioner in a quiet and relaxed condition; the detailed methods were as follows: ① The participants were asked to maintain an orthopnea position after taking about 5 minutes' rest, then the watch or accessories from the left wrist were removed. ② Extend forward the left hand naturally so as to make sure the forearm is in the same height level with the heart. Place the left wrist on the pulse diagnosis pillow with palms facing up in order to make the beating place of the radial artery fully exposed. ③ The “Guan” pulse was determined at the pulse point of the radial artery inside the styloid process of the radius by the testers' middle finger pulp. And the pressure probe of the pulse measurement device was fastened on the “Guan” pulse position. ④ The probe was rotated to pressurize and when the pressure stabilized, the pulse images' recording started. The pulse images were recorded at 50g, 125g, and 175g pressures, respectively, and the pulse images under optimal pressure were selected automatically by the pulse measurement device. ⑤ The classical time-domain variables *h*_1_, *h*_4_, *h*_5_, *t*_1_, *t*_3_, *t*_4_, *t*_5_, *h*_3_/*h*_1_, *h*_4_/*h*_1_, *w*/*t* were calculated using the software included in the DDMX-100 type pulse measurement device.

**Figure 2:**
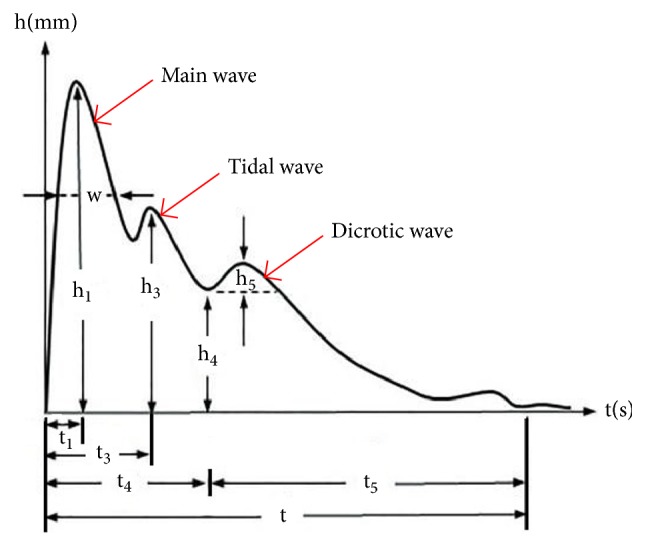
The time-domain variables of pulse signal. Notes: this pulse signal sample is a triple-peak waveform, which includes the main wave, the tidal wave, and the dicrotic wave. The y-axis is the amplitude of the pulse signal whose unit is millimetre (mm). The x-axis is the time whose unit is second (s).

As the most commonly used pulse wave form analysis in TCM [30], the classical time-domain variables are described through some characteristic points on the pulse wave (see Figure 2). The implications of the classical time-domain variables are as follows [31].

  h_1_: height of the main wave, namely, the height from the peak of the main wave to the baseline of the pulse chart.
  h_3_: height of the tidal wave, namely, the height from the peak of the tidal wave to the baseline of the pulse chart.
  h_4_: height of the dicrotic notch, namely, the height from the valley bottom of the dicrotic notch to the baseline of the pulse chart.
  h_5_: height of the dicrotic wave, namely, the height from the peak of the dicrotic wave to the baseline parallel line at the valley bottom of the dicrotic notch.
  t_1_: time distance between the starting point of pulse chart and the main wave peak, which corresponds to the rapid ejection period of the left ventricle.
  t_3_: time distance between the starting point of pulse chart and the tidal wave peak.
  t_4_: time distance between the starting point of pulse chart and dicrotic notch, which corresponds to the systole period of the left ventricle.
  t_5_: time distance between dicrotic notch and the ending point of pulse chart, which corresponds to the diastolic period of the left ventricle.
  t: time distance between the starting point and the ending point.
  w: width of main wave in its 1/3 height position.
  h_3_/h_1_: the ratio of h_3_ to h_1_.
  h_4_/h_1_: the ratio of h_4_ to h_1_.
  w/t: the ratio of w to t.

### 2.4. Statistical Analysis

If not otherwise stated, data are presented with mean±SD. In the continuous data, we used ANOVA multiple comparison test or T-test to compare differences among those three groups or between two groups for the normally distributed data, while Kruskal-Wallis ANOVA or a Mann–Whitney U test was for nonnormally distributed data and a Dunn-Bonferroni test for post hoc pairwise comparisons. In the categorical data, Pearson x^2^ test was used, and Bonferroni correction was used to adjust P value for the multiple comparisons.

To identify the association between radial pulse variables and arterial stiffness/related echocardiographic parameters, linear and stepwise regression analysis was performed using different models. Model 1 adjusted for age, sex, BMI, blood pressure, and pulse pressure, model 2 included model 1 and adjusted additionally for duration time of hypertension, history of taking antihypertensive drug, and the controlling condition of blood pressure. Model 3 included model 2 and adjusted additionally for arterial stiffness (PWV and ABI), which was only used to detect the association between pulse variables and related echocardiographic parameters.

For statistical analysis, P values are two sided and subject to a global significance level of 0.05, and they would be conducted in the IBM SPSS 21.0 (IBM Corporation, Armonk, NY, USA).

## 3. Results

### 3.1. The Baseline Characteristics of Studied Participants

The baseline characteristics of 159 participants are shown in Table 1. In the study cohort, 42 subjects met the health control criteria, 64 subjects were in the hypertension group, and the remaining 53 ones were diagnosed with hypertension heart disease. Age, BMI, pulse pressure, and Ba-PWV for all three studied groups were statistically different (p<0.05). In addition, the hypertension heart disease group had a poorer blood pressure control and longer duration time of hypertension than the hypertension group (p<0.05). What is more, except for the diminishing EF, all the remaining structural echocardiographic parameters including AAO, AODd, LVDs, LVDd, VST, PWT, and LAD were higher in the hypertension heart disease group than the other two groups (p<0.05).

As for the radial artery pulse wave variables, only h_5_ value of the three groups was statistically different (p<0.05). Besides, compared with health control group and the hypertension heart disease group, the hypertension group had the lowest h_5_ value. Moreover, we could see that h_1_ value might be the second obviously changed variables among those three groups (p=0.081), while more samples should be needed in the future to detect this difference.

### 3.2. The Association between Radial Artery Pulse Wave Variables and Arterial Stiffness

#### 3.2.1. The Correlation Study in the Total Studied Population

The association between radial artery pulse wave variables and arterial stiffness in the total studied population is shown in Table 2. Stepwise regression analysis using model 1 demonstrated that baPWV was independently associated with t_1_ value (*β* = -0.21, P<0.001), t_5_ value (*β* = -0.27, P<0.001), h_1_ value (*β* = 0.19, P=0.001), and h_3_/h_1_ value (*β* =0.24, P<0.001). ABI was independently associated with h_4_ value (*β* =0.19, P=0.026). Besides, after additional adjustment for duration time of hypertension, history of taking antihypertensive drugs, and the controlling condition of blood pressure (model 2), baPWV was still independently associated with t_1_ value (*β* = -0.21, P=0.001), t_5_ value (*β* = -0.28, P<0.001), h_1_ value (*β* = 0.19, P=0.001), and h_3_/h_1_ value (*β* =0.25, P<0.001), so did ABI with h_4_ value (*β* =0.19, P=0.022). The above results suggested that a higher baPWV was independently correlated with shorter t_1_ and t_5_ value but higher h_1_ and h_3_/h_1_ value, while ABI was positively associated with h_4_ value independent of the above adjusted risk factors.

#### 3.2.2. The Correlation Study in Each Different Group

The association between radial artery pulse variables and arterial stiffness in each different group is shown in Table 3. For all the three groups, only model 2 was used in stepwise regression analysis. In the health control group, baPWV was independently associated with t_3_ value (*β*=-0.31, P=0.014). In the hypertension group, baPWV was independently associated with h_1_ value (*β*=0.25, P=0.015). In the hypertensive heart disease group, t_3_ value (*β*=-0.42, P=0.001), t_5_ value (*β* =-0.31, P=0.006), and h_5_ value (*β*=-0.32, P=0.008) were the independent factors for baPWV, while for ABI, it was only associated with t_3_ value (*β*=-0.35, P=0.014) and h_4_ value (*β*=0.31, P=0.032) in the hypertensive heart disease group. The above results demonstrated that the premature appearance of tidal wave was a sign for a higher baPWV in the healthy old population. Besides, it also indicated that a higher main wave was associated with a higher baPWV in the subjects with hypertension. Moreover, premature appearance of tidal wave, the shortening of the diastolic period of the left ventricle, and the lowering of the dicrotic wave were all related with a higher baPWV in the hypertension subjects with target organ-damage to heart.

### 3.3. The Association between Radial Artery Pulse Variables and Echocardiographic Parameters

#### 3.3.1. The Correlation Study in the Total Studied Population

The association between radial artery pulse variables and echocardiographic parameters in the total studied population is shown in Table 4. Stepwise regression analysis using model 1 demonstrated that h_5_ value was the only radial artery pulse variable which independently associated with AAO (*β* =0.20, P=0.011), AODd (*β* = 0.21, P=0.006), VST (*β* = 0.17, P=0.035), PWT (*β* = 0.16, P=0.047), LVDs (*β* = 0.43, P<0.001), and LVDd (*β* =0.38, P<0.001), while t_1_ (*β* = -0.27, P=0.002), h_5_ (*β* =- 0.32, P<0.001), h_4_/h_1_ (*β* = 0.27, P=0.001), and w/t (*β* = -0.35, P<0.001) were the independent factors which affected the EF value (P<0.05). However, no correlation was found between radial artery pulse variables and LAD.

Besides, after additional adjustment for duration time of hypertension, history of taking antihypertensive drug, the controlling condition of blood pressure (model 2), and extra adjustment for baPWV and ABI (model 3), h_5_ value was still the most relevant factor which is associated with almost all the echocardiographic parameters especially for LVDs (*β* =0.48, P<0.001) and LVDd (*β* =0.40, P<0.001). In addition, LVDs were additionally associated with t_4_ value (*β* =0.15, P=0.039). However, for the EF value, h_4_ was the only independent factor. Therefore, those results indicated that, independent of the above adjusted risk factors, h_5_ and t_4_ were positively correlated with echocardiographic parameters including AAO, AODd, VST, PWT, LAD, LVDs, and LVDd; however, h_4_ value was positively associated with EF.

#### 3.3.2. The Correlation Study in Each Different Group

The association between radial artery pulse variables and echocardiographic parameters in each different group is shown in Table 5. For all the three groups, only model 3 was used in stepwise regression analysis. In the health control group, h_4_ was independently associated with AAO (*β* =-0.46, P=0.015) and AODD (*β* =-0.41, P=0.030). Besides, h_5_, t_1_, and w/t were all negatively associated with VST and PWT. No correlation was found for radial artery pulse wave variables with LAD, LVDs, LVDd, and EF. In the hypertension group, h_1_ was independently associated with PWT (*β* =0.34, P=0.016), w/t was independently associated with LAD (*β* =0.39, P=0.022), h_5_ was, respectively, related to LVDs (*β* =0.53, P<0.001), LVDd (*β* =0.41, P=0.003), and EF (*β* =-0.36, P=0.018), and h_4_/h_1_ was independently associated with LVDs (*β* =-0.24, P=0.043). In the hypertensive heart disease group, t_1_ was independently associated with AODd (*β* =-0.39, P=0.013) and LVDd (*β* =-0.38, P=0.034), h_5_ was independently related to LVDs (*β* =0.40, P=0.012), h_1_ (*β* =0.35, P=0.017) was independent factors for LVDd, and t_5_ was independently associated with EF (*β* =0.33, P=0.034). The above results indicated that, independent of the above adjusted risk factors, h_4_, h_5_, t_1_, and w/t value were negatively related to AAO, AODD, VST, and PWT in the healthy population, while in the hypertension population, the augmentation of h_1_, h_5_, and w/t and decrease of h_4_/h_1_ were hints for a higher PWT, LAD, LVDs, and LVDd and a lower EF. Moreover, in the population with hypertensive heart disease, the shortening of t_1_ and t_5_ and the increase of h_1_ and h_5_ suggested the increase of AODD, LVDs, and LVDd and the decrease of EF.

## 4. Discussion

Pulse condition is a state of pulse throbbing which described the feeling of a finger's palpation. For the blood vessels of human run through the whole body to connect both the internal organs and the external skins, it often provides comprehensive information on the visceral functions, qi and blood, Yin and Yang, which can be used for diagnostic purposes [23]. Pulse diagnosis, as one of the four classical methods (inspection, auscultation and olfaction, inquiry and palpation) in traditional Chinese medicine, plays an important role in the diagnosis of diseases.

By observing the three groups (Table 1), our data showed a statistical difference in age, BMI, pulse pressure, medicine-taking history, blood pressure control condition, duration time of hypertension, and baPWV (p<0.05). In addition, more abnormal cardiovascular structure changes occurred in the hypertensive heart disease group compared with the remaining two groups (p<0.05). However, compared with above cardiovascular factors, the radial pulse wave variables were less sensitive to the comparison among the three groups of individuals. This might be caused by the fact that radial arterial pulse parameters are actually affected by multiple factors which included arterial elasticity, blood pressure, and cardiovascular function rather than certain unitary factor. As a result, a high standard deviation that in some cases reaches 30% was observed, which indicated a high degree of data dispersion. In our study, the variable with the most obvious change in radial artery pulse was h_5_ value, followed by h_1_ value. This suggests that the decrease in dicrotic wave and increase in main wave can be detected before the cardiac remodelling and dysfunction. This is partly in line with the previous researches which reported a lower dicrotic wave and a higher main wave in the hypertension group compared with the health control [16, 17, 32].

The stepwise regression analysis in association between radial artery pulse variables and arterial stiffness in the total studied population showed that shortening both in the rapid ejection period and in the diastolic period of the left ventricle, as well as ascending of the main wave and h_3_/h_1_, might be associated with arterial stiffness. Besides, a lower dicrotic notch might be associated with abnormal patency of peripheral occlusive arterial disease. In previous subjective studies, it revealed that the superficial, strong, and fast pulse images were related to higher PWV. By application of objectified pulse diagram parameters, similar results are obtained from our study [19, 20], while in the correlation study in each different group, we still found that a decrease in t_3_ and h_5_ value was also independently related to increase of baPWV under the same cardiac condition. In one word, arterial stiffness was suggested by increase of the main wave (h_1_) and the ratio between h_3_ and h_1_ (h_3_/h_1_), the premature appearance of the tidal wave (t_3_), the shortening of rapid ejection time (t_1_), and diastolic time of left ventricle (t_5_) as well as decrease of the dicrotic wave (h_5_).

In the total studied population, the stepwise regression analysis in association between radial artery pulse variables and echocardiographic parameters showed that the increase of h_5_ was the most relevant factor which associated with all the echocardiographic parameters except for the EF value, especially for its associations with LVDs and LVDd. This result was partly in line with the previous research which reported that a larger systolic and diastolic left ventricular dimensions, lower EF, and poorer thickening properties of the left ventricular posterior wall had been observed in subjects whose dicrotic wave was greater than 20% of the main wave via carotid pulse tracing than those who not [33]. However, for the EF value, h_4_ was the only independent factor. Through the correlation study in each different group, we found that the decrease of h_4_ value was also independently related with increase of AAO (diameter of ascending aorta) and AODd (aorta sinus department diameter) in the healthy population. Besides increase of h_1_ was independently associated with increase of PWT (left ventricular posterior wall thickness) in hypertension population and LVDd (left ventricular end-diastolic diameter) in population with hypertensive heart disease. Decrease of t_1_ was independently related with the increase of VST and PWT in healthy population and the increase of AODd and LVDd in population with hypertensive heart disease. At last, t_5_ was positively related with EF in population with hypertensive heart disease.

Based on the above results, in the radial artery pulse variables, decrease of t_1_, t_5_ and increase of h_1_, h_3_/h_1_ were statistically associated with arterial stiffness in the total population without regard to the cardiac factors and blood pressure. However, decrease of t_3_ and h_5_ was also hints for arterial stiffness when the cardiac function and blood pressure were under the same condition. In terms of echocardiographic parameters, our data showed that the height of dicrotic wave indicated by h_5_ was the most relevant pulse variable. The increase of h_5_ was associated with the decrease of EF but the increase of all the remaining echocardiographic parameters especially for LVDs and LVDd. The normal dicrotic wave has been generally ascribed to a reflected wave from the recoil of the blood column against the closed aortic valve [34, 35]. Therefore the primary determinants of the dicrotic wave are contractile state of the ventricle, the competence of the aortic valve, the distensibility of the arterial system, and the systemic blood pressure which acts indirectly by modifying arterial distensibility [36]. It was reported that the normal dicrotic wave is diminished or lost with age, hypertension, arteriosclerosis, and diabetes mellitus [31, 33]. However impaired cardiac function with a low cardiac output was also related to occurrence of a larger dicrotic wave [36, 37]. In our study, a significant lower dicrotic wave was observed in the hypertension group compared with the other two; this may be due to the systemic blood pressure which acts indirectly by modifying arterial stiffness. However, a higher dicrotic wave occurred in the hypertensive heart disease group than the remaining two groups. The main reason for that is the impaired cardiac function in spite of the modified arterial distensibility. The semilunar valves are so delicately constructed that they readily respond when the pressure on one side rises above that on the other. As soon as the aortic pressure rises above the ventricle the valves close. We assume that under the same level of arterial stiffness, when the contractile state of ventricle is impaired, then the ventricular pressure is much less than the aortic pressure, which eventually augment the vibration caused by aorta closure; as a result, a higher dicrotic wave would be detected at an impaired cardiac function.

To our knowledge, the current study is the first quantitative study to define the exact associations between radial artery pulse wave variables and cardiovascular conditions (assessed by arterial stiffness measurements and echocardiography) in hypertension. This study would provide an experimental basis for cardiovascular pathophysiological mechanism of radial artery pulse wave variables.

Several limitations merit discussion. Firstly, the study sample was limited to the patients diagnosed only with systematic hypertension and/or hypertensive heart disease at the age of 45-75 years. A more large-scale study should be carried out in the future to include more cardiovascular risk factors like age, BMI, glucose, lipid, etc. Secondly, as this was a cross-sectional study, we only investigate the history of taking antihypertensive drugs rather than the concrete type of the current antihypertensive drugs. Further longitudinal studies should be conducted to determine whether these changes of radial artery pulse waves evolve in the clinical outcomes of the hypertension as well as the impact of different concrete type of the current antihypertensive drugs on the radial pulse wave changes. Thirdly, this study is conducted by the objective radial artery pulse variables at “Guan” position; a subjective pulse palpation performed by TCM experts should be conducted in the future so as to get a thorough understanding of the pulse changes in hypertension.

## 5. Conclusion

In this study, we found radial artery pulse wave variables in association with the arterial stiffness and echocardiographic changes. For all the time-domain variables of wrist pulse, the decrease of t_1_, t_3_, t_5_, h_5_ and the increase of h_1_, h_3_/h_1_ might suggest the arterial stiffness. In addition, a higher h_5_ was the most relevant factor which affected structural echocardiographic variables.

## Figures and Tables

**Table 1:** Characteristics of the study subjects.

	Health control	Hypertension	Hypertension heart disease	P value
	(n=42)	(n=64)	(n=53)	
**Demographic factors**				
Sex (male)	45.24%	45.31%	62.26%	0.130
Age in years	58.74±6.32	62.52±6.53	63.85±8.49	**0.003**
BMI (kg/m2)	23.16±2.35	23.23±2.77	25.27±2.37	**<0.001**
systolic blood pressure (mmHg)	124.36±11.01	135.97±14.95	147.42±14.92	**<0.001**
diastolic blood pressure (mmHg)	73.00±8.02	78.34±8.18	83.08±10.37	**<0.001**
Pulse pressure (mmHg)	51.36±7.38	57.63±11.09	64.34±11.87	**<0.001**
Heart rates (beats/min)	72.02±11.52	76.02±12.98	72.38±15.2	0.221
Medicine-taking history	None	95.31%	92.45%	**<0.001**
Poor blood pressure control	None	40.62%	66.03%	**<0.001**
duration time of hypertension (years)	None	7.29±4.95	9.66±6.72	**<0.001**

**Arterial stiffness measurements**				
Ba-PWV (cm/s)	1473.17±182.55	1689.03±285.59	1791.53±291.77	**<0.001**
ABI	1.08±0.09	1.11±0.07	1.12±0.07	0.067

**Echocardiographic parameters**				
AAO (mm)	29.88±3.06	31.38±2.79	34.32±3.38	**<0.001**
AODd (mm)	29.95±2.98	30.77±2.7	32.85±3.04	**<0.001**
LVDs (mm)	29.02±2.59	28.44±3.32	32.15±3.28	**<0.001**
LVDd (mm)	45.60±3.43	45.42±3.92	49.96±3.99	**<0.001**
VST (mm)	8.30±0.62	8.52±0.85	10.25±2.09	**<0.001**
PWT (mm)	8.30±0.62	8.38±0.77	10.25±2.06	**<0.001**
LAD (mm)	33.60±2.96	34.97±2.84	40.98±2.86	**<0.001**
EF (%)	65.21±4.44	66.7±4.38	63.98±5.92	**0.006**
E/A⩽1	52.38%	75.00%	83.02%	**0.003**

**Changes in cardiovascular structure or function**				
LV active relaxation delay	45.24%	68.75%	26.42%	**<0.001**
LV diastole dysfunction	None	None	52.83%	**<0.001**
Thickening in the LV wall	None	None	5.66%	**0.035**
Thickening in the base of IVS	None	None	33.96%	**<0.001**
LA Enlargement	None	None	64.15%	**<0.001**
LV Enlargement	None	None	7.55%	**0.015**
RA Enlargement	None	None	5.66%	**0.035**
Broadening in the AAO or AS	None	None	11.32%	**0.003**

**TCM pulse variables**				
t_1_(s)	0.14±0.03	0.14±0.03	0.13±0.03	0.479
t_3_(s)	0.25±0.02	0.25±0.03	0.24±0.02	0.211
t_4_(s)	0.34±0.03	0.34±0.04	0.34±0.04	0.921
t_5_(s)	0.50±0.11	0.49±0.13	0.52±0.13	0.339
h_1_(mm)	15.09±4.27	16.76±4.84	16.57±4.64	**0.081**
h_4_(mm)	6.46±1.74	6.72±2.02	6.84±2.21	0.650
h_5_(mm)	-0.06±0.19	-0.30±0.45	0.04±0.45	**<0.001**
h_3_/h_1_	0.77±0.13	0.77±0.13	0.77±0.14	0.970
h_4_/h_1_	0.44±0.09	0.41±0.09	0.42±0.09	0.209
w/t	0.23±0.02	0.24±0.04	0.23±0.04	0.472

Data shown are mean±SD, or proportions (in percentages). LV: left ventricle; LA: left atrium; RA: right atrium; IVS: interventricular septum; AAO: ascending aorta; AS: aorta sinus.

**Table 2:** The association between radial artery pulse wave variables and arterial stiffness in the total studied population.

Dependent	Variables entered	R^2^	B	95% CI of B	*β*	P value
PWV	Model 1					
	t_1_(s)	0.61	-2131.21	[-3314.17, -948.25]	-0.21	<0.001
	t_5_(s)		-642.11	[-939.5, -344.72]	-0.27	<0.001
	h_3_/h_1_		535.32	[243.35,827.29]	0.24	<0.001
	h_1_(mm)		12.09	[4.77,19.40]	0.19	0.001
	Model 2					
	t_1_(s)	0.62	-2130.99	[-3330.02, -931.96]	-0.21	0.001
	t_5_(s)		-653.68	[-953.48, -353.88]	-0.28	<0.001
	h_3_/h_1_		544.88	[250.76,839.01]	0.25	<0.001
	h_1_(mm)		12.06	[4.71,19.41]	0.19	0.001

ABI	Model 1					
	h_4_(mm)	0.05	0.01	[0.00,0.01]	0.19	0.026
	Model 2					
	h_4_(mm)	0.09	0.01	[0.00,0.01]	0.19	0.022

Stepwise regression included the radial artery pulse wave variables and age, sex, BMI, blood pressure and pulse pressure (model 1), and, additionally, use of duration time of hypertension, history of taking antihypertensive drugs, and the controlling condition of blood pressure (model 2). The partial regression coefficient B, 95% CI of B, and standard regression coefficient *β* are shown for parameters who entered into the model.

**Table 3:** The association between radial artery pulse variables and arterial stiffness in each different group.

Group	Dependent	Variables entered	R^2^	B	95% CI of B	*β*	p
Health control	PWV	t_3_(s)	0.58	-2693.12	[-4796.66, -589.57]	-0.31	0.014
	ABI	/	/	/	/	/	/

Hypertension	PWV	h_1_(mm)	0.58	14.89	[2.99,26.8]	0.25	0.015
	ABI	/	/	/	/	/	/

Hypertensive heart disease	PWV	t_3_(s)	0.61	-5075.69	[-7901.75, -2249.63]	-0.42	0.001
		t_5_(s)		-711.14	[-1210.25, -212.03]	-0.31	0.006
		h_5_(mm)		-208.61	[-359.6, -57.63]	-0.32	0.008
	ABI	t_3_(s)	0.38	-0.99	[-1.77, -0.21]	-0.35	0.014
		h_4_(mm)		0.01	[0,0.02]	0.31	0.032

Stepwise regression included the radial artery pulse wave variables and age, sex, BMI, blood pressure, and pulse pressure and, additionally, use of duration time of hypertension, history of taking antihypertensive drug, and the controlling condition of blood pressure (model 2). The partial regression coefficient B, 95% CI of B, and standard regression coefficient *β* are shown for parameters who entered into the model.

**Table 4:** The association between radial artery pulse variables and echocardiographic parameters in the total studied population.

Dependent	Variables entered	R^ 2^	B	95% CI of B	*β*	P value
AAO	Model 1					
	h_5_(mm)	0.21	1.71	[0.40,3.02]	0.20	0.011
	Model 2					
	h_5_(mm)	0.27	1.9	[0.62,3.19]	0.23	0.004
	Model 3					
	h_5_(mm)	0.29	1.9	[0.63,3.17]	0.23	0.004

AODd	Model 1					
	h_5_(mm)	0.24	1.58	[0.45,2.71]	0.21	0.006
	Model 2					
	h_5_(mm)	0.28	1.79	[0.67,2.91]	0.24	0.002
	Model 3					
	h_5_(mm)	0.31	1.79	[0.68,2.89]	0.24	0.002

VST	Model 1					
	h_5_(mm)	0.23	0.63	[0.04,1.22]	0.17	0.035
	Model 2					
	h_5_(mm)	0.24	0.69	[0.09,1.28]	0.18	0.024
	Model 3					
	h_5_(mm)	0.25	0.69	[0.10,1.28]	0.18	0.023

PWT	Model 1					
	h_5_(mm)	0.26	0.6	[0.01,1.19]	0.16	0.047
	Model 2					
	h_5_(mm)	0.24	0.59	[0.00,1.19]	0.16	0.050
	Model 3					
	h_5_(mm)	0.26	0.6	[0.01,1.19]	0.16	0.047

LAD	Model 1					
	/	/	/	/	/	/
	Model 2					
	h_5_(mm)	0.39	1.47	[0.05,2.89]	0.15	0.042
	Model 3					
	h_5_(mm)	0.39	1.47	[0.05,2.90]	0.15	0.042

LVDS	Model 1					
	h_5_(mm)	0.34	3.6	[2.41,4.80]	0.43	<0.001
	Model 2					
	h_5_(mm)	0.37	4	[2.78,5.23]	0.48	<0.001
	t_4_(s)		15.88	[0.78,30.97]	0.15	0.039
	Model 3					
	h_5_(mm)	0.38	4.01	[2.79,5.23]	0.48	<0.001
	t_4_(s)		16.13	[0.80,31.46]	0.15	0.039

LVDd	Model 1					
	h_5_(mm)	0.32	3.87	[2.38,5.37]	0.38	<0.001
	Model 2					
	h_5_(mm)	0.35	4.11	[2.62,5.60]	0.40	<0.001
	Model 3					
	h_5_(mm)	0.39	4.11	[2.66,5.56]	0.40	<0.001

EF	Model 1					
	h_5_(mm)	0.23	-3.9	[-5.99, -1.80]	-0.32	<0.001
	t_1_(s)		-48.98	[-79.41, -18.55]	-0.27	0.002
	w/t		-51.22	[-77.41, -25.04]	-0.35	<0.001
	h_4_/h_1_		15.77	[6.14,25.41]	0.27	0.001
	Model 2					
	h_4_(mm)	0.13	0.51	[0.11,0.90]	0.20	0.013
	Model 3					
	h_4_(mm)	0.14	0.50	[0.09,0.90]	0.20	0.016

AAO: diameter of ascending aorta; AODd: aorta sinus department diameter; VST: interventricular septal thickness; PWT: left ventricular posterior wall thickness; LAD: left atrial diameter; LVDs: left ventricular end-systolic diameter; LVDd: left ventricular end-diastolic diameter; EF: ejection fraction.

Stepwise regression included the radial artery pulse wave variables and age, sex, BMI, blood pressure, and pulse pressure (model 1) and, additionally, use of duration time of hypertension, history of taking antihypertensive drug, and the controlling condition of blood pressure (model 2), and, moreover, use of PWV and ABI (model 3). The partial regression coefficient B, 95% CI of B, and standard regression coefficient *β* are shown for parameters who entered into the model.

**Table 5:** The association between radial artery pulse variables and echocardiographic parameters in each different group.

Groups	Dependent	Variables entered	R^2^	B	95% CI of B	*β*	p
Health control	AAO	h_4_(mm)	0.24	-0.82	[-1.47, -0.17]	-0.46	0.015
	AODD	h_4_(mm)	0.22	-0.71	[-1.34, -0.07]	-0.41	0.030
	VST	h_5_(mm)	0.51	-2.29	[-3.35, -1.22]	-0.71	<0.001
		t_1_(s)		-12.62	[-20.29, -4.95]	-0.56	0.002
		w/t		-8.95	[-17.46, -0.44]	-0.36	0.040
	PWT	h_5_(mm)	0.51	-2.29	[-3.35, -1.22]	-0.71	<0.001
		t_1_(s)		-12.62	[-20.29, -4.95]	-0.56	0.002
		w/t		-8.95	[-17.46, -0.44]	-0.36	0.040
	LAD	/	/	/	/	/	/
	LVDs	/	/	/	/	/	/
	LVDd	/	/	/	/	/	/
	EF	/	/	/	/	/	/

Hypertension	AAO	/	/	/	/	/	/
	AODd	/	/	/	/	/	/
	VST	/	/	/	/	/	/
	PWT	h_1_(mm)	0.32	0.05	[0.01,0.10]	0.34	0.016
	LAD	w/t	0.27	31.37	[4.69,58.05]	0.39	0.022
	LVDs	h_5_(mm)	0.42	3.95	[1.99,5.92]	0.53	<0.001
		h_4_/h_1_		-9.44	[-18.57, -0.31]	-0.24	0.043
	LVDd	h_5_(mm)	0.40	3.57	[1.23,5.90]	0.41	0.003
	EF	h_5_(mm)	0.23	-3.57	[-6.52, -0.63]	-0.36	0.018

Hypertensive heart disease	AAO	/	/	/	/	/	/
	AODd	t_1_(s)	0.49	-40.39	[-71.94, -8.85]	-0.39	0.013
	VST	/	/	/	/	/	/
	PWT	/	/	/	/	/	/
	LAD	/	/	/	/	/	/
	LVDs	h_5_(mm)	0.28	2.93	[0.69,5.17]	0.40	0.012
	LVDd	h_1_(mm)	0.35	0.30	[0.06,0.55]	0.35	0.017
		t_1_(s)		-51.31	[-98.42, -4.20]	-0.38	0.034
	EF	t_5_(s)	0.37	15.79	[1.22,30.36]	0.33	0.034

AAO: diameter of ascending aorta; AODd: aorta sinus department diameter; VST: interventricular septal thickness; PWT: left ventricular posterior wall thickness; LAD: left atrial diameter; LVDs: left ventricular end-systolic diameter; LVDd: left ventricular end-diastolic diameter; EF: ejection fraction.

Stepwise regression included the radial artery pulse wave variables and age, sex, BMI, blood pressure and pulse pressure, use of duration time of hypertension, history of taking antihypertensive drug, the controlling condition of blood pressure, and, moreover, use of PWV and ABI (model 3). The partial regression coefficient B, 95% CI of B, and standard regression coefficient *β* are shown for parameters who entered into the model.

## Data Availability

The datasets generated and analyzed during the current study are not publicly available due to the confidentiality of the data, which is an important component of the National Key Technology R&D program of the 13th Five-Year Plan (No. 2017YFC1703301) in China, but are available from the corresponding author on reasonable request.

## Abbreviations

PWV: Pulse wave velocity
ABI: Ankle brachial index
AAO: Diameter of ascending aorta
AODd: Aorta sinus department diameter
VST: Interventricular septal thickness
PWT: Left ventricular posterior wall thickness
LAD: Left atrial diameter
LVDs: Left ventricular end-systolic diameter
LVDd: Left ventricular end-diastolic diameter
EF: Ejection fraction.
